# Pre-Transplant Dual-Energy X-ray Absorptiometry (DXA)-Derived Body Composition Measures as Predictors of Treatment Outcomes and Early Post-Transplant Complications in Patients with Multiple Myeloma (MM) Treated with Autologous Hematopoietic Stem Cell Transplantation (AutoHSCT)

**DOI:** 10.3390/jcm13195987

**Published:** 2024-10-08

**Authors:** Paula Jabłonowska-Babij, Diana Jędrzejuk, Maciej Majcherek, Agnieszka Szeremet, Magdalena Karasek, Bartłomiej Kuszczak, Krzysztof Kujawa, Milena Sitkiewicz, Marcin Landwójtowicz, Tomasz Wróbel, Maciej Tomasiewicz, Anna Czyż

**Affiliations:** 1 Department and Clinic of Hematology, Blood Neoplasms and Bone Marrow Transplantation, Wroclaw Medical University, 50-367 Wroclaw, Poland; maciej.majcherek@umw.edu.pl (M.M.); agnieszka.szeremet@umw.edu.pl (A.S.); magdalena.karasek@student.umw.edu.pl (M.K.); bartkuszczak@gmail.com (B.K.); milsit7@gmail.com (M.S.); tomasz.wrobel@umw.edu.pl (T.W.); maciej.tomasiewicz@usk.wroc.pl (M.T.); a.czyz@umw.edu.pl (A.C.); 2 Department and Clinic of Endocrinology, Diabetology, and Isotope Therapy, Wroclaw Medical University, 50-367 Wroclaw, Poland; diana.jedrzejuk@umw.edu.pl (D.J.); marcin.landwojtowicz@umw.edu.pl (M.L.); 3 Statistical Analysis Centre, Wroclaw Medical University, 50-367 Wroclaw, Poland; krzysztof.kujawa@umw.edu.pl

**Keywords:** body composition, multiple myeloma, autologous hematopoietic stem cell transplantation, progression-free survival, infections

## Abstract

**Background/Objectives**: Changes in muscle mass and bone density are common in multiple myeloma (MM) patients. Dual-energy X-ray absorptiometry (DXA) offers precise, non-invasive insights into a patient’s physical condition before autologous stem cell transplantation (autoHSCT). This study examines how pre-transplant body composition impacts treatment outcomes and early complications in MM patients undergoing autoHSCT. **Methods**: This study is a single-center, retrospective analysis of patients with MM who were treated with first or second autoHSCT and underwent DXA pre-transplant between 11 August 2019 and 12 June 2024. **Results**: We conducted a study of pre-transplant body composition in 127 patients with MM. Among them, 108 (85%) qualified for first autoHSCT, while 19 (15%) qualified for a second. The median age of the patients was 64 years (range 50–73). In the Cox proportional hazards regression conducted in the group of women, Total Body %Fat was a statistically significant predictor for progression-free survival (PFS) (HR = 0.07, 95% CI = 0.01,0.6, *p* = 0.0157). In the Mann–Whitney U test conducted on males, Lean Mass/Height^2^ and Appen. Lean Height^2^ were statistically significant predictors of early infections after autoHSCT (Z = 1.98, *p* = 0.0473 and Z = 2.32, *p* = 0.0204, respectively). In males, Fat Mass/Height^2^ was a significant predictor of non-infectious toxicity related to treatment (Z = −1.98, *p* = 0.0476). **Conclusions**: In women, higher levels of adipose tissue initially appear to exert a protective effect; however, this benefit diminishes over time, with greater fat mass eventually correlating with an increased risk of disease progression. In men, muscle mass has been identified as a significant predictor of early infection risk post-autoHSCT. Furthermore, our findings indicate that an increased amount of adipose tissue in men is statistically associated with a higher risk of non-infectious treatment-related toxicity. These conclusions highlight the critical need for further investigation into the role of body composition.

## 1. Introduction

Multiple myeloma (MM), also known as plasma cell myeloma (PCM), is a clinically heterogeneous malignancy that accounts for 2% of newly diagnosed cancer cases worldwide and slightly more than 10% of all hematological malignancies [1,2]. The disease is characterized by the excessive and uncontrolled proliferation of malignant plasma cells, which leads to severe complications, including anemia, skeletal abnormalities, renal impairment, immune system dysfunction, and disruptions in protein metabolism [3,4].

Despite significant advances in treatment methods, including the introduction of immunotherapy over the past two decades, high-dose chemotherapy (HDC) supported by autologous hematopoietic stem cell transplantation (autoHSCT) remains an integral part of the initial treatment for MM patients. It is also increasingly used for patients with refractory/relapsed (R/R) disease [5,6]. AutoHSCT demonstrates high efficacy in prolonging overall survival (OS) and progression-free survival (PFS), as well as improving the quality of life for patients. However, it remains associated with pre- and post-transplant complications, especially considering that this procedure is predominantly performed in elderly patients (the median age at MM diagnosis is 65 years, according to the World Health Organization (WHO), who often have multiple comorbidities and other health issues [2,7,8,9,10].

Changes in muscle mass and decreased bone density are common complications in patients with MM, often related to the disease, corticosteroid therapy, and anticancer treatment. Dual-energy X-ray absorptiometry (DXA) provides precise, non-invasive, and cost-effective measurements that offer valuable insights into the patient’s physical condition, directly impacting quality of life and therapy tolerance [11,12,13,14].

Although bone density results are an integral part of DXA findings, this publication focuses on the analysis of body composition. Bone changes are well documented in the literature and are recognized as significant predictors of treatment outcomes. It is well known that patients with MM frequently experience osteolytic lesions, osteopenia, and osteoporosis, which are often complicated by skeletal-related events (SREs) and are consequently associated with poorer OS, PFS, and a decline in quality of life [15,16,17,18,19,20].

Therefore, the primary objective of this study was to investigate the impact of pre-transplant body composition on treatment outcomes and the risk of early complications (infections and non-infectious toxicity of conditioning treatment) after autoHSCT in patients with MM. Specific objectives included examining whether there are significant differences in pre-transplant body composition results between patients before the first autoHSCT and before the second autoHSCT. Additionally, this study aimed to determine whether pre-transplant body composition is correlated with selected laboratory parameters, including C-reactive protein (CRP), white blood cells (WBCs), hemoglobin (Hb), total cholesterol, lactate dehydrogenase (LDH), aspartate aminotransferase (AST), alanine transaminase (ALT), albumin, neutrophils, lymphocytes, platelets (PLT), total protein, IgM, IgG, IgA, creatinine, triglycerides, and total calcium.

This study was conducted not merely to explore the potential associations and correlations between pre-transplant body composition and auto-transplant outcomes in patients with MM, but more importantly, to provide therapeutic teams—including physicians, physiotherapists, and other healthcare professionals—with practical guidelines and clinical implications. These insights can be applied in their work to enhance therapeutic approaches and preventive measures, ultimately contributing to the development of a more effective and holistic care protocol for patients in this group.

## 2. Materials and Methods

This study is a retrospective analysis of patients with MM who were treated with first or second autoHSCT and underwent DXA before autoHSCT between 11 August 2019 and 12 June 2024. Only patients who met the inclusion criteria and did not meet any of the exclusion criteria (see below) were included in the study.

Basic information was collected from the patients paper and electronic medical records, including gender, age, subtype of MM, date of diagnosis, date of autoHSCT, number of autoHSCTs, comorbidities, type of chemotherapy, number of cycles, and response to induction treatment (according to the International Myeloma Working Group (IMWG) criteria). To thoroughly describe and analyze the impact of other clinical predictors, we also included clinical stage according to the International Staging System for Multiple Myeloma (ISS) and the Durie–Salmon staging system (D-S), number of osteolytic lesions, the Cumulative Illness Rating Scale (CIRS), Body Mass Index (BMI), and results of selected laboratory parameters, including CRP, WBC, Hb, total cholesterol, LDH, AST, ALT, albumin, neutrophils, lymphocytes, PLT, total protein, IgM, IgG, IgA, creatinine, triglycerides, and total calcium.

The final status of all patients was obtained from paper and electronic medical records.

For this purpose, two independent reviewers conducted a comprehensive review of existing publications, following the Preferred Reporting Items for Systematic Reviews and Meta-Analyses [21]. The reviewers searched PubMed, Cochrane Library, Scopus, Web of Science, Google Scholar, and Excerpta Medica Database (EMBASE) without any restrictions. Additionally, they manually searched reference lists of key studies and reviews. The last literature search was conducted on 19 August 2024. Consistent keywords were used across different databases, including the following: densitometry, plasma cell myeloma, multiple myeloma, autologous hematopoietic stem cell transplantation, body composition, and DXA.

The study was assessed based on specific inclusion and exclusion criteria (see below).

Inclusion criteria: (i) Subject is ≥50 years old; (ii) Subject is postmenopausal if female, defined as having ceased menstruation for at least 12 consecutive; (iii) Subject underwent DXA before autoHSCT and no later than one week prior to the autoHSCT; (iv) Subject underwent autoHSCT; (v) Subject has availability of complete DXA and clinical data.

Exclusion criteria: (i) Subject does not have complete DXA or clinical data; (ii) Subject is diagnosed with other disease that may affect body composition (e.g., muscular dystrophies); (iii) Subject has >2 autoHSCTs; (iv) Subject has undergone surgical procedures that could potentially affect DXA results (e.g., implantation of a prosthesis, vertebroplasty procedure).

For each patient included in the study, DXA (Horizon A, Hologic, Marlborough, MA, USA, 2017) was performed no later than one week before the autoHSCT. To ensure accurate results, calcium administration and calcium-containing supplements were discontinued for all patients 24 h prior to the DXA.

For this analysis, pre-transplant DXA-derived body composition measures—including Total Body %Fat, Fat Mass/Height^2^, Android/Gynoid Ratio, %Fat Trunk/%Fat Legs, Trunk/Limb Fat Mass Ratio, Estimated VAT Mass (Est. VAT Mass), Lean Mass/Height^2^, and Appendicular Lean Mass/Height^2^ (Appen. Lean Height^2^)—were utilized to obtain more meaningful and relative values by normalizing the results in relation to other clinical factors. These measures provide a more nuanced evaluation by accounting for individual differences in body composition and other variables, thereby improving the accuracy and comparability of findings across the study population. This approach ensures that the results reflect not only absolute measurements but also insights adjusted for key clinical characteristics, offering a more comprehensive understanding of the data [22,23,24].

Continuous variables with normal distribution are presented as the mean with standard deviation (SD), and median (Me) with minimum and maximum values were used to present variables with non-normal data distribution. Categorical variables are presented as counts with percentages.

The one-variable Cox proportional hazards regression analysis was used to calculate hazard ratios (HRs) as measures of the association between OS and PFS and pre-transplant DXA-derived body composition measures. The assumptions of (1) proportional hazards and (2) linearity in the relationship between the log hazard and the predictors were checked using Schoenfeld residuals tests and Martingale residuals plots, respectively. When the assumption of a proportional hazard was not met, the interactions between the given predictor and log(time) were added to the model, where “time” was PFS. The log function was used based on shape of the relationships; this was only applied to females (and PFS only), as in males the assumption of proportional hazard was met in all the models. HRs were used to assess the risk factors associated with the time to outcome development. All ratios are presented with corresponding 95% confidence intervals (CIs). The predictive ability of the models was assessed with the use of the concordance index (C-index) [25].

The Mann–Whitney U test (with continuity correction) was applied to pre-transplant DXA-derived body composition measures between the groups with and without the early complications (infections and non-infectious toxicity of conditioning treatment) after autoHSCT and to compare pre-transplant DXA-derived body composition measures between the patients before the first autoHSCT and the patients before the second autoHSCT.

Spearman correlation analysis was conducted to investigate the relationships between pre-transplant DXA-derived body composition measures and selected laboratory parameters in males and females separately.

Non-parametric tests were used as the data distribution of many variables deviated from a normal distribution in ways that were statistically significant (in the Lilliefors test).

OS was calculated from the time of autoHSCT to death (event) or the end of the observation period (see below).

PFS was calculated from the time of autoHSCT to disease progression or the end of the observation period, 19 July 2024.

Early post-transplant complications were assessed in the first 100 days after transplant.

All statistical analyses were performed using Statistica 13.3 (TIBCO Software Inc., Palo Alto, CA, USA, 2017) and the R-package “survival”, which was used to perform the Cox regression models (including assumptions checking) [26].

*p* < 0.05 was considered statistically significant.

**Ethics**

According to the Declaration of Helsinki, this study was approved by the Wroclaw Medical University Ethics Committee. Approval number KB-482/2024; date of approval: 30 July 2024.

Collected data did not include any personally identifiable information; therefore, informed consent from the patients was not required.

## 3. Results

### 3.1. Patients Characteristics and Diagnostic Approach

A total of 141 patients who met the inclusion criteria were screened in this study. According to the exclusion criteria, 14 patients were excluded. Therefore, a total of 127 (52 men and 75 female) patients were included in the present study (Table 1). A mean age was 62.84 ± 5.08 (SD) (range: 50–73). All patients were diagnosed with MM (according to WHO classification). Prior to the start of the standard conditioning regimen (melphalan) before autoHSCT, each participant’s height and weight were measured, based on which the BMI (kg/m^2^) was calculated. The mean value of BMI was 27.1 ± 4.46 (SD) (range: 17.4–39.3). Median survival in this study was not calculated as in both OS and PFS, survival was higher than 0.5 (it amounted to 0.679 and 0.746, respectively).

### 3.2. DXA-Derived Body Composition Measures vs. Treatment Outcomes

**Figure 1:**
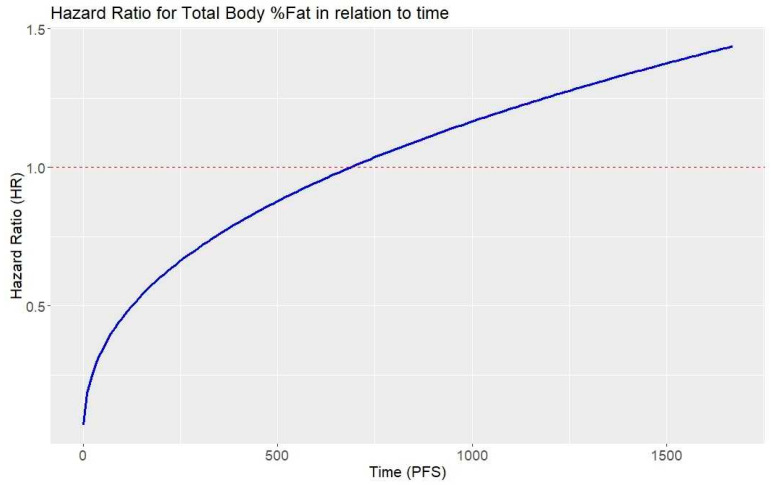
Hazard ratio for Total Body %Fat in relation to time.

In the Cox proportional hazards regression performed in the group of women, Total Body %Fat was a statistically significant predictor for PFS (Table 2). In this case, the HR of the predictor is <1, meaning that, initially, the higher the value of this variable, the lower the risk of recurrence. However, the HR of the interaction between this predictor and time is >1, indicating that over time, the HR increases (Figure 1). Eventually, this rise may outweigh the initial protective effect of the variable, and from a certain point, the Total Body %Fat variable may start to promote recurrence.

In females, none of the variables presented statistical significance for OS. However, Lean Mass/Height^2^ and Appen. Lean Mass/Height^2^ showed marginal non-significance, and the HR < 1 suggests that higher values of these predictors may potentially be associated with a lower risk of death and longer OS. Similarly, in males, none of the predictors achieved statistical significance for OS or PFS. However, for PFS, Total Body %Fat demonstrated marginal non-significance, and its HR > 1 indicates that higher values of this predictor may be associated with an increased risk of progression, resulting in shorter PFS (Table 2).

### 3.3. DXA-Derived Body Composition Measures vs. Early Complications after autoHSCT

The Mann–Whitney U test performed in males demonstrates that Lean Mass/Height^2^ and Appendicular Lean Mass/Height^2^ are statistically significant in the context of early infection risk after autoHSCT. Among the other predictors in males, none presented statistical significance, although Android/Gynoid Ratio approached marginal non-significance. In the female group, none of the predictors showed statistical significance; however, Total Body %Fat and Fat Mass/Height^2^ approached marginal non-significance. Regarding non-infectious toxicity related to conditioning treatment, only Fat Mass/Height^2^ in the male group was statistically significant (Table 3).

### 3.4. DXA-Derived Body Composition Measures vs. Number of AutoHSCTs

None of the pre-transplant DXA-derived body composition measures reached statistical significance in the context of comparing patients divided into two groups—women and men—before the first and second autoHSCT (Table 4).

### 3.5. DXA-Derived Body Composition Measures vs. Selected Laboratory Parameters

In males, an elevated ALT level was associated with an increase in Fat Mass/Height^2^, Lean Mass/Height^2^, and Appen. Lean Height^2^. Similarly, elevated IgM levels corresponded with a higher Android/Gynoid Ratio. Higher LDH levels were associated with an increase in Fat Mass/Height^2^ and Lean Mass/Height^2^. Elevated IgA levels were associated with a higher %Fat Trunk/%Fat Legs and Trunk/Limb Fat Mass Ratio. Furthermore, elevated hemoglobin (Hb) levels were correlated with a higher %Fat Trunk/%Fat Legs. Elevated IgG levels were inversely associated with Est. VAT Mass, meaning that higher IgG levels were linked to lower Est. VAT Mass (Table 5).

In females, elevated CRP levels were associated with an increase in Total Body %Fat, Fat Mass/Height^2^, %Fat Trunk/%Fat Legs, and Est. VAT Mass. Similarly, higher LDH levels were correlated with increased Total Body %Fat and Fat Mass/Height^2^. Elevated triglyceride levels were linked to increases in Total Body %Fat, Fat Mass/Height^2^, %Fat Trunk/%Fat Legs, and Est. VAT Mass. Furthermore, elevated platelet (PLT) counts were associated with higher a %Fat Trunk/%Fat Legs and Trunk/Limb Fat Mass Ratio. Higher IgG levels were associated with an increase in Lean Mass/Height^2^ and Appen. Lean Height^2^ (Table 6).

## 4. Discussion

Parameters such as muscle mass, fat mass, and their distribution can influence both treatment response and the risk of complications arising from anticancer therapy [27].

The results of this retrospective study, conducted on a group of MM patients prior to autoHSCT provide insights into the potential impact of body composition on treatment outcomes and the occurrence of early post-transplant infectious as well as non-infectious toxicity related to the conditioning treatment administered before autoHSCT.

Due to differences in muscle mass, fat mass, and their distribution between women and men, the study population was divided into two subgroups [28].

In women, the percentage of body fat, represented by Total Body %Fat, was found to be a statistically significant predictor for PFS. The HR values presented suggest that a higher fat percentage may initially offer protection against disease progression, which aligns with some previous literature on the protective role of body fat in certain conditions [29,30,31]. However, the interaction between body fat percentage and time indicates that over time, the HR increases, which may diminish the protective effect of this variable and, in the long term, contribute to a higher risk of disease progression. This phenomenon can be explained on several levels. Body fat can influence metabolism and hormonal balance, which may directly impact the overall health of patients in remission following cancer treatment and autoHSCT [32,33]. In the initial period following treatment, body fat may serve as an energy reserve, potentially supporting the body’s regenerative processes [34,35]. However, excess body fat can, over time, lead to inflammation, which may increase the risk of disease progression and metabolic complications [36,37,38]. The increase in HR over time highlights the dynamic nature of body composition’s impact on cancer progression, suggesting a strong need for further research to better understand the underlying mechanisms involved.

Interestingly, although none of the predictors for OS and PFS showed statistically significant associations in men, Total Body %Fat demonstrated marginal non-significance for PFS, with an HR >1, suggesting that higher levels of this variable may be linked to a greater risk of progression and thus a shorter PFS. This finding aligns with studies indicating that increased fat mass in men may act as a risk factor for the progression of certain cancers [39,40,41].

Moreover, for OS in women, although none of the variables presented statistical significance, the muscle mass indices adjusted for height, such as Lean Mass/Height^2^ and Appen. Lean Mass/Height^2^, showed marginal non-significance. An HR <1 suggests that higher values of these variables may be potentially associated with a lower risk of death and longer OS. Research on the relationship between muscle mass and OS is increasingly being discussed in the scientific literature. There is evidence that lean body mass, particularly muscle mass, has a significant impact on metabolic health, disease resistance, and survival across various populations, including cancer patients, individuals with cardiovascular diseases, and the general elderly population [42,43,44,45].

Sarcopenia, defined as the loss of skeletal muscle mass and function, has been recognized as an independent risk factor for increased mortality, particularly in postmenopausal women who are more susceptible to muscle loss due to hormonal changes. This condition has been linked to a higher risk of death, as the decline in muscle mass and strength significantly affects overall health, mobility, and resilience to disease [46,47,48]. Studies such as those conducted by Surow, A., et al. (2021) and Zakaria, HM., et al. (2020) have demonstrated that low muscle mass is strongly associated with poorer survival outcomes. These findings are consistent with our observations regarding Lean Mass/Height^2^ and Appen. Lean Mass/Height^2^, where greater muscle mass appears to have a protective effect, while a deficiency in muscle mass may lead to worse survival rates [49,50].

The amount of lean body mass adjusted for height, namely Lean Mass/Height^2^ and Appen. Lean Mass/Height^2^, was also found to be statistically significant in assessing the risk of early infections following autoHSCT, this time specifically in the male cohort. As previously mentioned, muscle mass, or the lack thereof, plays a crucial role in metabolic health, which in turn supports immune system function [51,52,53]. Maintaining adequate muscle mass can enhance the body’s ability to combat infections, especially during periods of physiological stress, such as stem cell transplant. In the context of autoHSCT, muscle mass serves as an essential energy reserve, and preserving mobility and physical activity can lead to a stronger immune response and lower the risk of infectious complications after transplantation. Our findings align with existing studies that suggest maintaining proper muscle mass, particularly in the limbs, can help reduce inflammation and support recovery following intensive medical procedures [54,55,56,57].

In the male group, it was also observed that the Android/Gynoid Ratio was close to statistical significance in relation to the risk of early infections post-transplantation. The results suggest that a higher value for this predictor may potentially increase the risk of infections after autoHSCT. This result aligns with current knowledge, which indicates that fat accumulation in the abdominal region, known as android fat, is strongly associated with chronic inflammation in the body and an increased risk of conditions such as diabetes and cardiovascular diseases [58,59]. This type of fat is more metabolically active and produces a higher amount of pro-inflammatory cytokines compared to fat located in the hips and thighs, known as gynoid fat [60,61]. A higher Android/Gynoid ratio can therefore lead to a weaker immune response and an increased risk of infections after autoHSCT [62,63].

In the male group, it was also observed that the Fat Mass/Height^2^ index, which represents fat mass adjusted for height, was statistically significant in relation to the risk of non-infectious toxicity associated with treatment. The results suggest that a higher fat mass is associated with an increased risk of non-infectious complications, which aligns with existing research indicating that excess fat can lead to inflammation, insulin resistance, and other metabolic disorders. These conditions may deteriorate the overall health of patients, particularly in the context of medical procedures, thereby increasing the likelihood of post-transplant complications [64,65,66].

In the female group, none of the variables reached statistical significance in relation to the occurrence of early infectious complications or non-infectious toxicity associated with treatment. However, Total Body %Fat and Fat Mass/Height^2^ were close to significance concerning the risk of infection. The results suggest that a higher fat mass in women may be potentially associated with an increased risk of infection after autoHSCT. An elevated level of body fat can lead to chronic inflammation and reduce the body’s immune efficiency, which raises the likelihood of infections. Additionally, excess fat is linked to hormonal imbalances and affects the immune system, decreasing its ability to fight off various pathogens [67,68,69].

In our study, we compared women with women and men with men who were being prepared for their first autoHSCT (i.e., those who had not previously undergone the procedure) with patients who had already undergone one autoHSCT and were being prepared for their second. The results of the Mann–Whitney U test did not reveal significant differences between these groups in terms of body composition. This suggests that autoHSCT may not have as significant an impact on body composition as one might expect. There are studies available in the literature that also suggest the impact of autoHSCT on body composition may be limited and more associated with short-term treatment effects rather than long-lasting changes [70].

Finally, the results reveal several significant associations between specific body composition metrics and selected laboratory parameters, which may be important in the context of preparing MM patients for autoHSCT.

In men, elevated ALT levels were associated with increases in both fat mass and lean body mass. This relationship may suggest that liver function alterations influence lipid and protein metabolism, which in turn affects body composition. It has been reported in the literature that liver dysfunction, including elevated ALT levels, can lead to metabolic changes such as visceral obesity. This has been confirmed in studies involving patients with chronic metabolic disorders [71,72,73,74]. LDH, frequently used as a marker of MM progression and disease aggressiveness, was also found to correlate with higher levels of both fat and lean body mass. Current research indicates that elevated LDH levels may contribute to increased muscle catabolism and alterations in fat distribution [75,76].

Similarly to men, higher levels of LDH and CRP were associated with an increase in adipose tissue in the group of women. CRP, as an indicator of inflammation, may contribute to the accumulation of visceral fat and alterations in fat distribution throughout the body [77]. Elevated triglyceride levels in this group were associated with an increase in adipose tissue, which may indicate lipid-related metabolic disturbances. Studies suggest that in cases of chronic inflammation and cancers such as MM, higher lipid levels may promote fat accumulation, particularly in the visceral region [78,79].

## 5. Conclusions

The results of our study suggest that body composition, including muscle and fat mass as well as their distribution, may play a significant role in predicting treatment outcomes and the risk of early infectious or non-infectious toxicity related to treatment in patients with MM prior to autoHSCT. In women, a higher level of adipose tissue initially appears to have a protective effect; however, over time, this effect diminishes, and a greater amount of fat tissue may lead to an increased risk of disease progression. In men, although no statistically significant results were obtained, a higher fat mass showed a trend toward being associated with shorter PFS.

Pre-transplant lean body mass adjusted for height showed marginal non-significance regarding OS in women, suggesting that higher muscle mass may potentially offer a protective effect on the OS of MM patients post-autoHSCT.

Furthermore, the findings indicate that muscle mass in men is a significant predictor of early infection risk after autoHSCT, emphasizing the importance of maintaining adequate muscle mass as an energy reserve and as support for immune system function. These results underscore the critical role of muscle preservation in improving both recovery and immune resilience in post-transplant patients.

Our findings also suggest that excess adipose tissue, particularly in the android region, may potentially increase the risk of early infections and non-infectious complications related to treatment following autoHSCT in men. This highlights the importance of body fat distribution as a factor in post-transplant outcomes and the potential need for targeted interventions to mitigate these risks.

Ultimately, these results underscore the need for further research into the impact of body composition on treatment outcomes, as well as the role that muscle and fat mass, along with their distribution, play in the preparation and recovery process for MM patients post-autoHSCT. Although autoHSCT does not appear to significantly affect long-term changes in body composition, pre-transplant body composition may serve as an important predictor of early post-transplant complications. This highlights the potential importance of considering body composition in patient management strategies to improve recovery and treatment success.

## 6. Limitations

It is important to acknowledge, however, that this study has certain limitations, primarily related to its design. First, the data collected and used in the analysis may be incomplete or inaccurate due to gaps and/or errors in documentation. The research team has, of course, drawn from multiple sources of information to ensure the highest possible level of accuracy. Second, our study is a single-center study, which may limit the generalizability of the findings to other settings. To address this issue, we have compared our data with the existing scientific literature to confirm their broader applicability.

## Figures and Tables

**Table 1:** Patients’ characteristics and treatment details.

Characteristic	Number (%)
Total number of pts	127 (100)
Me age (years)	64
Gender	
Male	52 (41)
Female	75 (59)
Type of MM	
IgG	76 (60)
IgA	22 (17)
IgD	3 (2)
Light chain	26 (21)
ISS	
I	26 (20)
II	30 (24)
III	41 (32)
Unk	30 (24)
D-S	
I (a or b)	5 (4)
II (a or b)	11 (9)
III (a or b)	63 (49)
Unk	48 (38)
Number of previous therapy lines	
1	94 (74)
2	26 (20)
3 or more	7 (6)
Number of autoHSCTs	
1	108 (85)
2	19 (15)
Status for response before autoHSCT	
CR	43 (34)
VGPR	47 (37)
PR	35 (27)
PD	2 (2)

Abbreviations: **Me**, median; **MM**, multiple myeloma; **ISS**, the International Staging System for Multiple Myeloma; **D-S**, the Durie–Salmon staging system; **autoHSCT**, auto stem cell transplantation; **CR**, complete response; **VGPR**, very good partial response; **PR**, partial response; **PD**, progression disease.

**Table 2:** The CoxPHR of treatment outcomes (OS and PFS) on pre-transplant DXA-derived body composition measures.

Variable	Males					Females—Predictors					Females—Interactions Predictor Time				
	HR	CI Lower	CI Upper	*p* Value	C-Index	HR	CI Lower	CI Upper	*p* Value	C-Index	HR	CI Lower	CI Upper	*p* Value	C-Index
Overall survival															
Total Body %Fat	1.05	0.92	1.19	0.4538	0.58	0.99	0.86	1.15	0.9116	0.46					
Fat Mass/Height^2^	0.98	0.85	1.13	0.7925	0.48	0.90	0.69	1.19	0.4699	0.56					
Android/Gynoid Ratio	2.40	0.03	181.99	0.691	0.52	0.10	0.00	18.86	0.3917	0.58					
%Fat Trunk/%Fat Legs	1.44	0.01	220.08	0.8867	0.49	0.04	0.00	66.78	0.4035	0.53					
Trunk/Limb Fat Mass Ratio	2.45	0.15	40.54	0.5304	0.54	0.04	0.00	5.11	0.1945	0.63					
Est. VAT Mass	1.00	1.00	1.00	0.2113	0.61	1.00	1.00	1.00	0.5466	0.58					
Lean Mass/Height^2^	0.90	0.71	1.15	0.4105	0.56	0.61	0.37	1.02	0.0583	0.71					
Appen. Lean Height^2^	0.75	0.43	1.31	0.3123	0.57	0.40	0.14	1.09	0.0736	0.72					
Progression-free survival															
Total Body %Fat	1.16	0.99	1.37	0.0736	0.72	0.07	0.01	0.60	0.0157		1.51	1.07	2.12	0.019	0.73
Fat Mass/Height^2^	1.02	0.91	1.14	0.7546	0.68	0.98	0.59	1.64	0.942		0.99	0.95	1.03	0.681	0.70
Android/Gynoid Ratio	33.34	0.29	3830.91	0.1474	0.69	0.83	0.01	85.56	0.937		0.99	0.97	1.01	0.345	0.69
%Fat Trunk/%Fat Legs	103.49	0.38	28,197.19	0.1049	0.67	1.17	0.01	212.40	0.953						0.44
Trunk/Limb Fat Mass Ratio	9.05	0.25	327.93	0.2293	0.62	1.18	0.04	34.41	0.922		0.99	0.97	1.01	0.245	0.70
Est. VAT Mass	1.00	1.00	1.01	0.1069	0.65	1.00	1.00	1.01	0.776		0.99	0.96	1.01	0.306	0.69
Lean Mass/Height^2^	1.10	0.82	1.46	0.5229	0.56	1.03	0.73	1.46	0.847		0.99	0.97	1.01	0.253	0.69
Appen. Lean Height^2^	1.31	0.72	2.39	0.3726	0.60	1.10	0.50	2.42	0.817		0.99	0.97	1.01	0.24	0.69

**Table 3:** Results of the Mann–Whitney U Test comparing pre-transplant DXA-derived body composition measures with early complications (infections and non-infection toxicity) after autoHSCT.

	Males		Females	
Variable	Z	*p* Corrected	Z	*p* Corrected
	Infections			
Total Body %Fat	0.01	0.9921	1.71	0.0873
Fat Mass/Height^2^	0.80	0.4220	1.83	0.0674
Android/Gynoid Ratio	1.71	0.0880	0.34	0.7332
%Fat Trunk/%Fat Legs	−0.47	0.6410	−0.31	0.7546
Trunk/Limb Fat Mass Ratio	−1.46	0.1449	−1.04	0.2958
Est. VAT Mass	0.35	0.7286	1.39	0.1658
Lean Mass/Height^2^	1.98	0.0473	1.22	0.2219
Appen. Lean Height^2^	2.32	0.0204	1.52	0.1294
	Non-infection toxicity			
Total Body %Fat	−1.05	0.2943	0.78	0.4325
Fat Mass/Height^2^	−1.98	0.0476	0.77	0.4424
Android/Gynoid Ratio	−0.36	0.7218	0.93	0.3524
%Fat Trunk/%Fat Legs	−0.60	0.5506	0.35	0.7298
Trunk/Limb Fat Mass Ratio	0.54	0.5901	0.86	0.3880
Est. VAT Mass	−1.36	0.1751	0.31	0.7553
Lean Mass/Height^2^	−1.69	0.0904	0.91	0.3641
Appen. Lean Height^2^	−1.41	0.1574	0.25	0.8022

**Table 4:** Results of the Mann–Whitney U Test comparing pre-transplant DXA-derived body composition measures with the number of autoHSCTs.

	Males		Females	
Variable	Z	*p* Corrected	Z	*p* Corrected
Total Body %Fat	0.77	0.4405	−0.88	0.3770
Fat Mass/Height^2^	0.40	0.6878	−0.63	0.5297
Android/Gynoid Ratio	−0.71	0.4800	−0.77	0.4387
%Fat Trunk/%Fat Legs	−0.07	0.9480	−1.64	0.1009
Trunk/Limb Fat Mass Ratio	0.20	0.8450	−1.00	0.3162
Est. VAT Mass	0.86	0.3909	−0.42	0.6753
Lean Mass/Height^2^	−0.76	0.4470	0.20	0.8412
Appen. Lean Height^2^	−0.74	0.4602	−0.50	0.6164

**Table 5:** Spearman correlation between pre-transplant DXA-derived body composition measures and selected laboratory parameters in males.

Variable	Total Body %Fat	Fat Mass/Height^2^	Android/Gynoid Ratio	%Fat Trunk/%Fat Legs	Trunk/Limb Fat Mass Ratio	Est. VAT Mass	Lean Mass/Height^2^	Appen. Lean Height^2^
Hb	0.06^0.6631^	0.11^0.4506^	0.19^0.1862^	0.30^0.0303^	0.16^0.2629^	0.04^0.7613^	0.15^0.2800^	0.22^0.1234^
WBCs	0.18^0.2050^	0.18^0.2077^	0.08^0.5634^	−0.23^0.0960^	−0.22^0.1143^	0.14^0.3075^	0.16^0.2686^	0.09^0.5175^
Neut	0.18^0.1921^	0.20^0.1526^	0.12^0.3879^	−0.14^0.3144^	−0.21^0.1445^	0.22^0.1238^	0.17^0.2228^	0.14^0.3054^
Limf	0.09^0.5192^	0.02^0.8798^	−0.07^0.6102^	−0.06^0.6633^	0.08^0.5721^	−0.14^0.3228^	−0.08^0.5738^	−0.16^0.2482^
Neut/Limf ratio	0.12^0.4083^	0.15^0.3003^	0.05^0.7108^	−0.10^0.4903^	−0.20^0.1462^	0.26^0.0609^	0.12^0.3907^	0.14^0.3187^
PLT	0.25^0.0688^	0.22^0.1216^	0.09^0.5280^	0.26^0.0585^	0.23^0.1058^	0.24^0.0913^	0.09^0.5334^	0.08^0.5867^
Total protein	−0.02^0.8938^	0.02^0.8906^	0.17^0.3107^	0.25^0.1205^	0.26^0.1031^	−0.08^0.6355^	0.10^0.5516^	0.07^0.6759^
CRP	0.19^0.1776^	0.13^0.3613^	0.00^0.9808^	−0.05^0.7469^	0.12^0.4059^	0.17^0.2307^	−0.02^0.8792^	−0.06^0.6610^
IgM	0.05^0.7654^	0.13^0.4214^	0.34^0.0367^	0.11^0.4944^	−0.06^0.7365^	−0.02^0.9247^	0.19^0.2527^	0.25^0.1222^
IgG	−0.16^0.2584^	−0.18^0.2175^	0.01^0.9679^	0.08^0.6065^	0.02^0.8857^	−0.30^0.0377^	−0.12^0.4028^	−0.10^0.5042^
IgA	−0.02^0.8998^	0.00^0.9990^	0.23^0.1581^	0.34^0.0341^	0.33^0.0449^	0.10^0.5434^	0.14^0.4031^	0.04^0.8097^
Alb	−0.02^0.8827^	0.01^0.9544^	0.26^0.0685^	0.02^0.9085^	−0.02^0.8854^	−0.01^0.9607^	0.09^0.5393^	0.10^0.4951^
Total chol	−0.09^0.5734^	−0.09^0.5782^	0.16^0.2948^	0.18^0.2545^	0.05^0.7362^	0.20^0.2062^	−0.04^0.8135^	0.04^0.8216^
AST	0.04^0.7663^	0.15^0.2809^	0.11^0.4477^	0.27^0.0503^	0.25^0.0689^	0.06^0.6475^	0.27^0.0534^	0.26^0.0616^
ALT	0.22^0.1202^	0.33^0.0187^	0.13^0.3403^	0.06^0.6888^	−0.01^0.9381^	0.17^0.2191^	0.40^0.0036^	0.44^0.0011^
Creatinine	−0.11^0.4510^	−0.08^0.5829^	0.17^0.2151^	0.02^0.8715^	−0.12^0.3906^	−0.15^0.2862^	−0.02^0.8758^	0.04^0.7956^
LDH	0.20^0.1467^	0.29^0.0379^	0.20^0.1512^	0.02^0.8776^	0.09^0.5393^	0.23^0.0989^	0.28^0.0431^	0.27^0.0505^
Triglycerides	−0.01^0.9606^	0.14^0.3689^	0.10^0.5378^	0.09^0.5644^	−0.12^0.4497^	0.20^0.2003^	0.26^0.0926^	0.30^0.0531^
Total calcium	−0.04^0.7696^	0.03^0.8338^	0.05^0.7415^	0.04^0.7799^	0.01^0.9294^	−0.07^0.6285^	0.16^0.2481^	0.23^0.1039^

Abbreviations: **Hb**, hemoglobin; **WBCs**, white blood cells; **Neut**, neutrophils; **Limf**, lymphocytes; **PLT**, platelets; **CRP**, C-reactive protein; **Alb**, albumin; **Total chol**, total cholesterol; **AST**, aspartate aminotransferase; **ALT**, alanine transaminase; **LDH**, lactate dehydrogenase.

**Table 6:** Spearman correlation between pre-transplant DXA-derived body composition measures and selected laboratory parameters in females.

Variable	Total Body %Fat	Fat Mass/Height^2^	Android/Gynoid Ratio	%Fat Trunk/%Fat Legs	Trunk/Limb Fat Mass Ratio	Est. VAT Mass	Lean Mass/Height^2^	Appen. Lean Height^2^
Hb	−0.13^0.2561^	−0.14^0.2348^	−0.02^0.8504^	−0.11^0.3455^	−0.05^0.6787^	−0.12^0.2992^	−0.02^0.8778^	−0.01^0.9088^
WBCs	0.01^0.9350^	0.02^0.8938^	−0.04^0.7448^	0.07^0.5733^	0.06^0.6111^	0.01^0.9248^	−0.04^0.7537^	−0.03^0.7893^
Neut	0.03^0.7752^	0.02^0.8502^	−0.07^0.5455^	0.02^0.8517^	0.04^0.7235^	−0.03^0.8008^	−0.06^0.6344^	−0.06^0.6156^
Limf	−0.06^0.6295^	−0.02^0.8400^	0.10^0.4036^	0.16^0.1662^	0.07^0.5592^	0.12^0.3095^	0.03^0.7682^	0.12^0.3204^
Neut/Limf ratio	0.11^0.3641^	0.10^0.4162^	−0.08^0.4786^	−0.06^0.5933^	−0.01^0.9514^	−0.08^0.5045^	0.00^0.9828^	−0.07^0.5557^
PLT	0.02^0.8854^	0.06^0.5881^	0.17^0.1461^	0.26^0.0255^	0.26^0.0236^	0.20^0.0789^	0.10^0.3865^	0.11^0.3444^
Total protein	0.13^0.3346^	0.16^0.2324^	0.12^0.4011^	0.13^0.3480^	0.02^0.8955^	0.09^0.5275^	0.18^0.1996^	0.25^0.0669^
CRP	0.33^0.0043^	0.29^0.0109^	0.10^0.3735^	0.23^0.0453^	0.17^0.1403^	0.25^0.0312^	0.12^0.3162^	0.09^0.4385^
IgM	0.08^0.5963^	0.13^0.4010^	0.01^0.9602^	−0.07^0.6526^	−0.11^0.4666^	−0.03^0.8304^	0.18^0.2445^	0.24^0.1109^
IgG	0.18^0.1342^	0.21^0.0761^	0.01^0.9088^	−0.04^0.7287^	−0.13^0.2882^	0.14^0.2258^	0.25^0.0313^	0.29^0.0121^
IgA	−0.16^0.2985^	0.01^0.9579^	−0.02^0.8972^	0.03^0.8632^	0.00^0.9992^	−0.04^0.8148^	0.27^0.0721^	0.21^0.1681^
Alb	0.22^0.0607^	0.13^0.2643^	0.13^0.2659^	0.10^0.3898^	0.03^0.8274^	0.05^0.7007^	−0.07^0.5413^	−0.04^0.7044^
Total chol	−0.01^0.9161^	−0.06^0.6884^	−0.07^0.6020^	−0.07^0.5969^	−0.12^0.3878^	−0.11^0.4306^	−0.09^0.5106^	0.10^0.4915^
AST	0.13^0.2826^	0.11^0.3479^	−0.03^0.8102^	−0.03^0.7766^	−0.04^0.7423^	−0.07^0.5602^	0.07^0.5468^	0.06^0.6212^
ALT	0.13^0.2699^	0.19^0.0958^	0.07^0.5277^	0.05^0.6401^	0.14^0.2150^	0.04^0.7513^	0.21^0.0747^	0.10^0.4039^
Creatinine	0.11^0.3683^	−0.02^0.8513^	−0.11^0.3269^	−0.06^0.6241^	−0.12^0.2983^	−0.14^0.2445^	−0.18^0.1286^	−0.15^0.2023^
LDH	0.26^0.0219^	0.25^0.0328^	0.06^0.5964^	0.20^0.0912^	0.06^0.6280^	0.11^0.3649^	0.11^0.3347^	0.22^0.0624^
Triglycerides	0.33^0.0132^	0.33^0.0153^	0.26^0.0556^	0.32^0.0181^	0.21^0.1154^	0.32^0.0180^	0.16^0.2433^	0.12^0.3866^
Total calcium	0.00^0.9695^	0.01^0.9559^	−0.01^0.9624^	0.08^0.5206^	0.04^0.7512^	−0.10^0.3904^	0.04^0.7655^	0.09^0.4358^

Abbreviations: **Hb**, hemoglobin; **WBCs,** white blood cells; **Neut**, neutrophils; **Limf**, lymphocytes; **PLT**, platelets; **CRP**, C-reactive protein; **Alb**, albumin; **Total chol**, total cholesterol; **AST**, aspartate aminotransferase; **ALT**, alanine transaminase; **LDH**, lactate dehydrogenase.

## Data Availability

The datasets generated and/or analyzed during the current study are available from the corresponding author on reasonable request.
